# The PTEN pathway in Tregs functions as a critical driver of the immunosuppressive tumor microenvironment and tolerance to apoptotic cells

**DOI:** 10.1186/2051-1426-3-S2-O19

**Published:** 2015-11-04

**Authors:** Madhav Sharma, Rahul Shinde, Tracy McGaha, Lei Huang, Rikke Holmgaard, Jedd Wolchok, Mario Mautino, Esteban Celis, Arlene Sharpe, Loise Francisco, Jonathan Powell, Hideo Yagita, Andrew Mellor, Bruce Blazar, David Munn

**Affiliations:** 1 Georgia Regents University, Augusta, GA, USA; 2 Memorial Sloan-Kettering Cancer Center, New York, NY, USA; 3 NewLink Genetics, Inc., Ames, IA, USA; 4 Harvard Medical School, Boston, MA, USA; 5 Johns Hopkins University School of Medicine, Baltimore, MD, USA; 6 Juntendo University School of Medicine, Tokyo, Japan; 7 University of Minnesota, Minneapolis, MN, USA; 8 Georgia Regents University Cancer Center, Augusta, GA, USA

## 

The tumor microenvironment is profoundly immunosuppressive, but exactly how this is coordinated and maintained remains poorly understood. We show that multiple transplantable and autochthonous mouse tumors actively elicit a population of highly suppressive regulatory T cells (Tregs) expressing the lipid phosphatase PTEN. These PTEN+ Tregs co-expressed PD-1, Foxp3, and high levels of Eos (*Ikzf4*). PTEN signaling acted to stabilize tumor-associated Tregs, maintaining their suppressor activity and preventing conversion into pro-inflammatory effector cells (“ex-Tregs”) in the face of inflammation. Mice with a targeted deletion of PTEN in Tregs (PTEN-Treg-KO mice) were healthy and fertile when young, but gradually developed lupus-like autoimmunity as they aged. Tumors implanted in young, healthy PTEN-Treg-KO mice were unable to create the normal immunosuppressive tumor microenvironment; instead, tumors were constitutively immunogenic, chronically inflamed, and could barely grow. In wild-type mice with large, pre-established tumors, pharmacologic inhibition of PTEN during the period following chemotherapy or adoptive immunotherapy caused a profound reconfiguration of the tumor microenvironment. The normally suppressive intratumoral Tregs became destabilized, and rapidly reprogrammed into pro-inflammatory “ex-Tregs” expressing IL-2, IL-17 and CD40L. The dominant APCs in the tumor changed from tolerogenic DCs expressing high levels of PD-L1, and were replaced by inflammatory myeloid DCs expressing high levels of CD86, MHC class II, IL-6 and IL-12. CD8+ effector T cells in the tumor, which had previously been unresponsive and PD-1+ (exhausted), became activated and expressed IFNγ and GzmB, and mediated tumor regression. Pharmacologic inhibition of PTEN was highly synergistic with conventional chemotherapy, allowing a single modest, normally ineffective dose of chemotherapy to trigger rapid tumor involution. This synergy was strictly immune-mediated, and was lost in the absence of host CD8+ T cells. In mice without tumors, identical PTEN+ Tregs were physiologically elicited by exposure to apoptotic cells; and PTEN-Treg-KO mice rapidly developed lupus-like autoimmunity when repeatedly challenged with apoptotic cells. The induction of PTEN+ Tregs by apoptotic cells was driven by indoleamine 2,3-dioxygenase (IDO) in the host, and was blocked by pharmacologic inhibition of IDO. Taken together, these data identify the PTEN pathway in Tregs as a potent immunosuppressive mechanism in tumors. PTEN+ Tregs controlled the downstream activation of inflammatory DCs and effector CD8+ T cells, and were part of the fundamental mechanism of tolerance to apoptotic cells. The PTEN pathway thus represents a potent, centrally-positioned immunosuppressive mechanism in tumors, which is amenable to pharmacologic inhibition and shows synergy with both adoptive immunotherapy and conventional chemotherapy.

